# The "not so good" thyroid cancer: a scoping review on risk factors associated with anxiety, depression and quality of life

**DOI:** 10.25122/jml-2022-0204

**Published:** 2023-03

**Authors:** Kyle Alexander, Sum-Yu Christina Lee, Stelios Georgiades, Constantina Constantinou

**Affiliations:** 1 Department of Basic and Clinical Sciences, University of Nicosia Medical School, Nicosia, Cyprus

**Keywords:** thyroid cancer, quality of life, depression, anxiety, cancer survivorship policy

## Abstract

The incidence of thyroid cancer has increased in recent years, leading to a growing number of survivors facing lifelong consequences. This scoping review investigated anxiety, depression, and quality of life (QoL) in thyroid cancer survivors compared to the general population, those with benign pathology, and survivors of other types of cancers. Moreover, we aimed to identify the risk factors associated with anxiety, depression, and QoL in thyroid cancer patients. A total of 727 articles were identified through PubMed, ProQuest, Cochrane, and Google Scholar databases, and 68 articles that met the criteria were selected for data extraction. Thyroid cancer survivors have a poorer QoL compared to the general population, population with benign pathology, and survivors of other types of cancer associated with worse clinical outcomes. The main risk factors are grouped into socioeconomic factors, disease-specific factors, management factors, comorbidities, and patient perceptions. Effective communication between the patient and the medical team and behavioral interventions may reduce these risks. Despite the common perception of thyroid cancer as a "good cancer," the findings of this review demonstrate the need to address the risk factors associated with increased anxiety, depression, and lower QoL in survivors.

## INTRODUCTION

Thyroid cancer (TC) develops from uncontrolled cell division in the tissues of the thyroid gland [1]. It is the most common type of endocrine cancer worldwide and ranks 9th in terms of overall cancer incidence [2]. In the UK, the incidence of TC is projected to increase from 6.5 to 11 cases per 100,000 individuals between 2014 to 2035 [3]. This type of cancer is more commonly diagnosed in women than men, with a prevalence that is approximately 2 to 3 times higher among women and is also one of the leading cancers among adolescents and young adults (AYAs) [4]. Despite its increasing incidence, TC has a very good prognosis, with a reported 5-year survival rate of 87% [5].

TC is usually asymptomatic in the early stages, but symptoms such as neck pain, hoarseness, and dysphagia may occur as the disease progresses. TC is diagnosed by physical examination, analysis of thyroid hormone levels, imaging, and biopsy [5]. The management of TC depends on the type and stage of cancer and patient preference. For low-risk TC, annual surveillance is recommended instead of immediate treatment [6]. The most frequent intervention is surgery, which includes either the complete removal of the thyroid (thyroidectomy), a partial thyroid removal (thyroid lobectomy), and/or lymph node dissection [7]. Radioactive iodine (RAI) is another treatment option, although it may cause side effects such as reduced fertility, salivary gland dysfunction, leukopenia, and eye inflammation [8]. Although less frequently used, other alternatives to surgery include external radiotherapy, chemotherapy, and targeted drug therapy [7]. In addition, patients often require lifelong levothyroxine for thyroid hormone supplementation and suppression of TSH-stimulated cancer growth [5].

Patients with cancer often experience significant psychological issues, including mood and anxiety disorders [9]. As a result, quality of life (QoL), which includes psychological, social, and spiritual well-being, has become an important consideration in the management of cancer survivors [10].

Due to the increasing incidence and improved prognosis of TC, a growing number of survivors are living with the effects associated with diagnosis and ongoing surveillance [11]. In addition, patients may experience depression as a comorbidity, with one study suggesting TC survivors have one of the highest depression prevalence among other cancer types [12].

This review aimed to compare the levels of anxiety, depression, and QoL in TC survivors with those of the general population, individuals with benign pathology, and survivors of other cancer types. Moreover, we aimed to investigate the risk factors associated with anxiety, depression, and low QoL in TC survivors.

## MATERIAL AND METHODS

In this scoping review, we adopted Arksey and O’Malley’s [13] scoping review framework, which includes the (1) identification of the research questions, (2) identification of relevant studies, (3) study selection, (4) charting the data, (5) collating, summarizing, and reporting the results.

### Step 1: Identifying the research questions

Our research questions were:

- What are the effects of TC on the anxiety, depression, and QoL of survivors, and how does this compare to the general population and survivors of other types of cancers?
- What risk factors are associated with increased anxiety and depression and lower QoL in TC survivors?
- Are there any protective factors associated with decreased anxiety and depression and improvement of QoL among TC survivors?

### Step 2: Searching for relevant studies

For this scoping review, we conducted an electronic search for articles written in English and published between 2015 and 2021, using Cochrane Library, ProQuest, PubMed, and Google Scholar databases. The search was conducted from June 2020 to December 2021. Our aim was to find primary studies that investigated and analyzed the links between thyroid cancer and QoL/depression/anxiety. The search was performed in the selected databases using the keywords "thyroid cancer AND (anxiety OR depression OR quality of life)". To ensure a comprehensive search and avoid duplication of results, a Boolean search was applied within each database. This method enabled us to get a large search output for eligible studies without using a multiple-stage search strategy.

#### Inclusion criteria

The primary inclusion criteria were articles containing the keywords: "thyroid cancer" and "quality of life" or "anxiety" or "depression". We limited our search to papers published in English between 2015-2021. We classified relevant papers based on several variables: title, keywords (listed by authors), journal, year of publication, country of study, relevant keywords extracted from the abstract/conclusion, and selection rationale.

#### Exclusion criteria

We excluded studies that focused on physical symptoms rather than anxiety and depression, studies that assessed only specific aspects of interventions, studies that evaluated QoL questionnaires rather than QoL itself, and studies that assessed healthcare personnel perceptions rather than patients’ perceptions. Additionally, we excluded studies that were not original journal articles, such as conference notes, narrative reviews, or studies that were not accessible in full text.

### Step 3: Selecting the studies

Two reviewers independently extracted data from the included studies. Finally, we summarized the studies based on their characteristics and reported a narrative synthesis of the results [13].

### Step 4: Charting the data

The articles were retrieved by authors KA and SL, and when there were discrepancies, a third reviewer, CC, was involved. A total of 727 articles were retrieved from four databases, and 60 duplicates were removed. The remaining articles were screened, and 522 articles that did not meet the inclusion criteria were removed, resulting in a final selection of 145 articles for more in-depth assessment.

### Step 5: Collating, summarizing, and reporting the results

We reviewed the results in an iterative manner, suggested refinements, and provided insights on the findings.

## RESULTS

**Figure 1:**
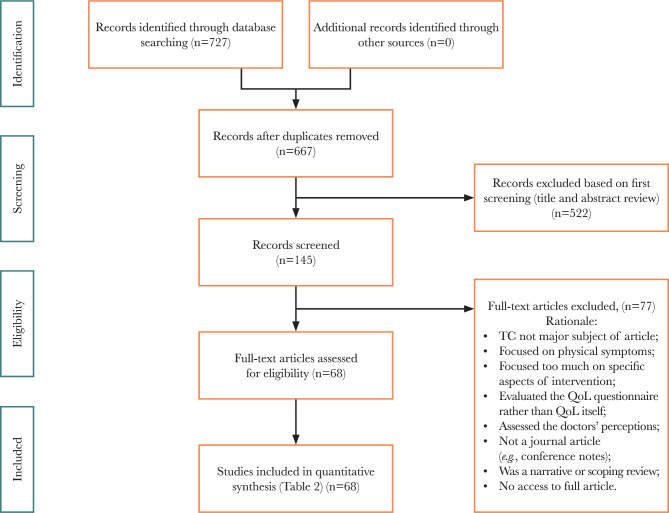
PRISMA for Anxiety, Depression, and QoL in TC patients and survivors. A scoping review from Google Scholar, Cochrane Library database, ProQuest, and PubMed. PRISMA Chart adapted from Moher *et al*. (2009).

The selection process is visualized using a PRISMA flow diagram (Figure 1). A total of 727 articles were retrieved from 4 separate databases, yielding 667 unique articles after deduplication. After reviewing the titles and abstracts, 522 articles did not meet the inclusion criteria and were excluded, leaving 145 articles included for full-text eligibility assessment. After thoroughly reviewing the 145 articles, 77 were removed based on the exclusion criteria, leaving a final selection of 68 papers (Figure 1). Each article was mapped to the three main concepts used to answer the three research questions in the methods section.

### TC survivors vs. the general population, population with benign pathology, and survivors of other types of cancers

Seventeen studies investigated depression, anxiety, or QoL in TC survivors versus the general population (Table 1) [14-30]. Several studies reported that QoL among TC survivors was generally lower than that of the general population [14-23, 29]. Goldfarb *et al*. [24] found that QoL was lower in female young adult TC survivors compared to their age-matched peers in the general population, whereas Mols *et al*. [25] reported that QoL was lower in AYA TC survivors compared to age-matched controls in the general population. In another study, TC survivors and controls scored similarly regarding QoL, anxiety, and depression. However, survivors reported physical problems, role limitations, and thyroid-specific symptoms [26].

**Table 1:** Studies comparing anxiety, depression, or QoL in TC patients compared to the general population and population with benign pathologies.

Study	Study details (design, population and methods)	Country study was conducted	Thyroid Cancer Concepts		
			Comparison of TC patients and survivors to the general population and population with benign pathologies	Key findings	Risk factor categories
Van Velsen *et al*. [14]	Prospective study on QoL in DTC patients (n=185). Questionnaires: ThyPRO, MFI-20, RAND-36	The Netherlands	The baseline QoL of TC patients was lower than the general population.	The quality of life (QoL) was found to be at its lowest during radioactive iodine (RAI) therapy, but it recovered with time. Factors that increased the risk of lower QoL were being female, younger in age, and having chronic hypoparathyroidism.	**Socioeconomic factors** Age: younger age associated with decreased QoL; Sex: being female associated with decreased QoL. **Disease-specific factors** Chronic hypoparathyroidism: associated with decreased QoL. **Management factors** RAI treatment: associated with lowest QoL score.
Wang *et al*. [15]	Cross-sectional study on QoL TC survivors (n=965) and comparison to controls (n=1790). Questionnaires: SF-36, EORTC QLQ-C30.	China	TC survivors have lower QoL compared to age/sex-matched controls within the same community.	Global QoL scores were associated with education, employment status, marital status, per capita disposable income, physical activity per week, fruit intake per day, and type of surgery.	**Socioeconomic factors** Education: being educated associated with higher QoL; Family status: being married associated with higher QoL; Financial status: higher income associated with higher QoL; Employment status: being employed associated with higher QoL.
Gou *et al*. [16]	Prospective observational study, where thyroidectomy (PTC) patients (n=186) had their QoL assessed, preoperatively and postoperatively, and compared to participants from the general population (control group). Questionnaire: SF-36 (Chinese version).	China	PTC patients had significantly lower scores than the general population in 7/8 domains of the HRQoL: role-physical, bodily pain, general health, vitality, social functioning, role-emotional, and mental health.	N/A	N/A
Hedman *et al*. [17]	Cross-sectional study on the long-term effects of QoL in DTC patients (n=279) compared to the general population. Questionnaires: SF-36 and a study-specific questionnaire.	Sweden	Compared to the general population, DTC patients had a significantly poorer QoL in 4 out of 8 domains (general health, vitality, social functioning, and mental health).	Patients who did not experience worry about cancer recurrence had a significantly better QoL in 5 out of 8 domains compared to those who had experienced recurrence or had worries about recurrence. Patients who reported that their TC led to a negative outlook on life had a significantly poorer QoL in all 8 domains. Patients with ≥2 comorbidities had a worse QoL in all 8 domains than those without.	**Comorbidities** Patients with ≥2 comorbidities: associated with decreased QoL; **Patients’ perceptions** Worry of recurrence: associated with decreased QoL; Negative view of life: associated with decreased QoL.
McIntyre *et al*. [18]	Cross-sectional study on DTC patients (n=82). Questionnaires: EQ-5D-3L and other.	UK	QoL of DTC patients was lower than the general population and other cancer (breast, colorectal, prostate) patients. Many reported symptoms of fatigue and depression.	N/A	N/A
Ali *et al*. [19]	Prospective case-control study on QoL in DTC patients (n= 89) and matched controls (n= 100). Questionnaire: SF-36.	Egypt	Baseline QoL was lower in DTC patients than healthy control in 6 out of 8 domains of SF-36.	QoL significantly decreased after levothyroxine withdrawal in DTC patients. The physical component summary (PCS) and component summary (CS) mean scores of QoL were significantly higher for patients <45 years of age when compared to older patients.	**Socioeconomic factors** Age: older age associated with higher risk of decreased QoL. **Management factors** Levothyroxine withdrawal: associated with higher risk of decreased QoL.
Li *et al*. [20]	Prospective study on QoL of DTC patients (n=174) after thyroidectomy compared against matched controls (from the general population) (n=174). Questionnaire: SF-36.	China	TC survivors scored significantly lower than the control population in 7 out of 8 domains of QoL.	TC survivors with no comorbidities had better QoL than those with ≥2 comorbidities.	**Comorbidities** Comorbidities: associated with lower QoL
Gamper *et al*. [21]	Prospective study on QoL of TC patients (n=284) and comparison with their before-treatment baseline and with age/sex-matched controls from the general population. Questionnaire: EORTC QLQ-C30.	Austria	QoL was decreased in almost all domains in TC patients compared to the general population.	Compared to the baseline (i.e., before treatment), the QoL of patients after treatment significantly increased, especially in domains such as role and emotional functioning, fatigue, pain, and dyspnoea. Some aspects of QoL also rose with exogenous TSH stimulation.	**Management factors** TSH stimulation: associated with improving some aspects of QoL
Haraj *et al*. [22]	Cohort study, evaluating QoL in DTC patients (n=124) and a control group of healthy subjects (n=124). Questionnaires: SF36, Hamilton anxiety, and Hamilton depression questionnaires.	Morocco	DTC patients' QoL was significantly impaired compared to healthy controls on all 3 questionnaires.	TMN stage, radioiodine therapy and dose, and the presence of metastases, were identified as the factors influencing QoL.	**Disease-specific factors** Cancer stage: Higher TNM stage and the presence of metastases associated with lower QoL **Management factors** Radioiodine therapy and higher dose: associated with lower QoL
Giusti *et al*. [23]	Longitudinal study evaluating QoL yearly in patients with a history of DTC, using the ThyPRO questionnaire from 2012 to 2016. All participants were under periodic surveillance and were compared to patients who had undergone thyroid surgery for a benign pathology (control group).	Italy	DTC and benign pathology patients had similar QoL, based on the ThyPRO questionnaire, except for scores on the hyperthyroid symptoms scale which were significantly higher in DTC patients.	QoL improved in the study group, over time. No significant differences in QoL between genders in DTC patients were reported, however, females showed a greater perception of illness.	**Socioeconomic factors** Sex: Females had increased perception of illness **Disease-specific factors** Years since diagnosis: increased time since disease presentation associated with a small improvement in QoL
Goldfarb *et al*. [24]	Cross-sectional study in which HRQOL was examined in TC survivors (n=1028), ≥17 years old. Mean scores from THYCA-QoL, MCS and PCS of SF-12v1, and derived SF-6D were compared between socio-demographic and clinical factors and to the general population.	USA	HRQoL is lower in female, young adult TC survivors compared to the normal (age-matched) population.	A higher level of education, female sex, unemployment, and having a comorbidity resulted in significantly higher THYCA-QoL scores (more complaints) and lower SF-6D scores (lower HRQOL; p < 0.05) in TC survivors.	**Socioeconomic factors** Sex: Female sex associated with lower QoL; Education: Higher education associated with lower QoL; Employment status: Unemployment associated with lower QoL. **Comorbidities** Comorbidity: associated with lower QoL
Mols *et al*. [25]	Cross-sectional study on QoL of TC survivors (n=293) using the EORTC QLQ-C30 and THYCA-QoL questionnaires. Results were compared against national normative data.	The Netherlands	Adolescent and young adult TC survivors scored worse than age-matched controls in physical, role, cognitive, and social functioning aspects of QoL. They also experienced more fatigue and financial problems.	Among TC survivors, adolescent and young adults scored better in physical function and interest in sex than the elderly and reported less autonomic and throat or mouth problems than those who are middle-aged.	**Socioeconomic factors** Age: Younger patients associated with increased QoL in various aspects compared to middle-aged and elderly patients
Nies *et al*. [26]	Cross-sectional case-control study on QoL, anxiety, and depression of adult survivors (n=76) of pediatric DTC and 56 controls using the SF-36 and HADS questionnaires.	The Netherlands	Survivors and controls scored similarly in most aspects, but survivors reported more physical problems and role limitations as a result of these. Survivors also reported thyroid-specific symptoms such as sensory disturbances and chilliness.	Risk factors for worse QoL were unemployment, more extensive disease, and more aggressive RAI treatment.	**Socioeconomic factors** Employment status: unemployment associated with decreased QoL **Disease-specific factors** Cancer stage: extensive disease associated with decreased QoL **Management factors** RAI treatment: more aggressive RAI treatment associated with decreased QoL
Yang *et al*. [27]	Cross-sectional study of levels and prevalence of anxiety in 529 thyroid tumor patients (139 TC patients and 390 benign thyroid tumor patients) compared to 419 controls using the State-Trait Anxiety Inventory.	China	TC patients had significantly higher anxiety levels than those with benign thyroid tumors, and those in the general population.	N/A	N/A
Choi *et al*. [28]	Prospective case-control cohort study on post-thyroidectomy TC patients (n=2609), matching incidence of a diagnosis of depression against matched-normative data.	South Korea	TC patients had a significantly higher incidence of depression in the first-year post-operation than the general population. No differences in the incidence of depression between TC patients and the general population more than 10 years after their operation.	Risk factors for depression included being a young adult (<44), female, living in urban areas, and having a low income.	**Socioeconomic status** Age: individuals younger than 44 at higher risk for depression; Sex: females at higher risk for depression; Living in urban areas: at higher risk for depression; Financial status: lower income associated with high risk of depression. **Disease-specific factors** Time lapse between surgery: Increased time since surgery associated with lower risk of depression
Chow *et al*. [29]	Retrospective matched-pair study on the effect of remnant radioiodine ablation on QoL in low-risk DTC patients (n=122 pairs) using the PROMIS-29 questionnaire.	USA	QoL of DTC patients was worse than the US general population.	QoL was not significantly different between surgery only and surgery plus RAI. Those with treatment-related complications reported significantly worse QoL than those without. RAI-associated xerostomia was also correlated with a worse QoL.	**Treatment-related factors** Complications: associated with lower QoL
Teliti *et al*. [30]	Comparative cross-sectional study evaluating sleep quality and its effects on QoL in DTC patients (n=119) compared to controls with benign thyroid pathology. QoL was measured using the Thyroid-specific patient-reported outcome (ThyPRO), the Billewicz scale (BS), and the ad-hoc visual analogic scale (VAS).	Italy	DTC patients had worse QoL overall compared to the control group with benign thyroid pathology.	In DTC patients, the levels of insomnia were significantly correlated with increased age and lower QoL.	**Comorbidities** Insomnia: associated with a worse QoL

Furthermore, studies reported that TC patients had higher levels of anxiety and depression compared to the general population [27, 28]. Two studies investigated the association between depression, anxiety, or QoL in TC patients versus those with benign pathologies [23, 30]. Giusti *et al*. [23] found that TC patients had no major differences in QoL but reported a significantly higher level of hyperthyroidism-related symptoms when compared to patients with benign thyroid pathology. Chow *et al*. found that TC patients experiencing complications from surgery and RAI had significantly worse QoL scores than those who did not [29]. In addition, a study by Teliti *et al*. [30] established that differentiated thyroid cancer (DTC) patients had worse QoL than patients with benign thyroid pathology. Overall, these studies suggest that TC survivors experience compromised QoL compared to the general population or those with benign pathology.

### TC patients vs. patients suffering from other types of cancer

Three studies examined the QoL, anxiety, and depression of TC patients compared to patients suffering from other types of cancers (Table 2) [31-33]. According to Applewhite *et al*. [31], TC survivors had a comparable QoL to the colon, glioma, and gynecological cancer survivors but a worse QoL than breast cancer survivors. Additionally, TC survivors reported inadequate support and having their diagnosis trivialized by healthcare professionals, as their cancer was commonly labeled as a "good cancer". Interestingly, one study [32] reported that TC survivors experienced higher levels of anxiety and depression compared to survivors of breast, colorectal, uterine, and prostate cancers or non-Hodgkin's lymphoma. The authors suggested that the continued surveillance of TC patients during the survivorship period contributes to anxiety and fear of cancer recurrence [32]. Aschebrook-Kilfoy *et al*. [33] found that TC patients had a lower QoL than patients with more invasive cancers, which is associated with worse disease outcomes. The study also highlighted that the psychological issues experienced during TC survivorship might be overlooked due to its good prognosis and suggested that the actual prognosis of cancer may have little correlation with its psychological impact [33]. Overall, the studies indicate that despite having a highly treatable "good" cancer, TC patients are not less likely to have a poor QoL and may even experience more prolonged periods of emotional distress compared to survivors of other cancers with worse prognoses.

**Table 2:** Studies comparing anxiety, depression, and QoL in TC patients and patients suffering from other types of cancer.

Study	Study details (design and population)	Country study was conducted	Thyroid cancer concepts		
			Comparison of TC patients and survivors to patients suffering from other types of cancer	Key findings	Risk factor categories
Applewhite *et al*. [31]	Cross-sectional study comparing QoL of 1174 TC patients from the NATCSS study with other cancers using the same QoL scoring system (QOL-CS).	USA	Self-reported QoL in TC was similar to or worse than other cancers with worse prognosis (colorectal, glioma, breast).	N/A	N/A
Goswami *et al*. [32]	Cross-sectional study on QoL of 1743 patients using the PROMIS-29 questionnaire. T-scores from it were compared with scores from patients suffering from other cancers and with US normative data.	USA	TC patients reported several worse QoL aspects (anxiety, depression, fatigue, sleep disturbance) than patients in other cancer cohorts (non-Hodgkin's lymphoma, breast, colorectal, uterine, prostate), but also reported less pain and more physical function.	N/A	N/A
Aschebrook-Kilfoy *et al*. [33]	Cross-sectional study of TC survivors (n=1174) and patients with other more invasive cancers using the City of Hope-QoL tool and open-ended questions.	USA	TC patients had a lower QoL compared to patients with more invasive cancers associated with worse disease outcomes (such as colorectal and breast cancers)	Risk factors of lower QoL in survivors were being female, younger age at diagnosis, and lower educational level.	**Socioeconomic factors** Age: younger patients at higher risk of decreased QoL; Sex: females at higher risk of decreased QoL; Educational level: patients with low educational level at higher risk of decreased QoL.

### Risk factors associated with anxiety, depression, and low QoL in TC patients

The current review has identified several risk factors associated with anxiety, depression, and low QoL in TC patients, which were grouped into the following categories: socioeconomic factors, disease-specific factors, management factors, comorbidities, and patient perceptions.

#### Socioeconomic factors

Several studies have reported an association between socioeconomic factors and anxiety, depression, or QoL in TC patients (Tables 1–3) [14, 19, 23, 25, 28, 33-50].

**Table 3:** Studies examining risk factors associated with lower QoL in thyroid cancer patients.

Study	Study details (design and population and methods)	Country study was conducted	Key findings	Risk factor categories
Papaleontiou [34]	Prospective study on QoL of DTC (n=349) patients. Questionnaires: SF-36 and study-specific questionnaire at diagnosis and one year later.	Sweden	At the time of follow-up, patients reported higher quality of life (QoL) scores compared to their initial diagnosis. However, it was observed that patients who had risk factors at diagnosis such as being over 50 years of age, having lower educational attainment, living alone, comorbidities, negative outlook towards life, and fear of recurrence had a decreased QoL score at follow-up.	**Socioeconomic factors** Age: >50 years old associated with decreased QoL; Education: lower educational attainment associated with decreased QoL; Family status: living alone associated with decreased QoL. **Comorbidities** Comorbidities: More comorbidities associated with decreased QoL **Patients’ perceptions** Negative view of life: associated with decreased QoL **Fear of recurrence:** associated with decreased QoL
Papaleontiou *et al*. [35]	Cross-sectional study on cancer-related worry in DTC patients (n=2215) using a worry-specific questionnaire.	USA	Patients worried most about recurrence, their family being at risk, impaired QoL, harm from treatments, and death. Higher levels of worry were associated with being female, of lower socioeconomic status, younger age, and of Hispanic or Asian ethnicity.	**Socioeconomic factors** Age: younger age associated with more worry; Sex: females associated with more worry; Ethnic background: Being Hispanic or Asian associated with more worry; Socioeconomic background: Lower social-economic background associated with more worry. **Patients’ perceptions** Fear of recurrence; Family at risk; Impaired QoL; Harm from treatments; Death.
Husson *et al*. [36]	Cross-sectional study of TC patients (n=293), comparing differences in illness perceptions between different age groups at the time of diagnosis. The HADS and B-IPQ questionnaires were used.	Netherlands	Adolescents and Young adults (AYAs) had more faith in their treatment, confidence in control of treatment, and understanding of illness. AYAs and older patients who believed their illness would continue for a long time reported more distress.	**Socioeconomic factors** Age: Younger individuals had more faith in their treatment, confidence about the control of their treatment, and greater understanding of their illness. **Patients’ perceptions** Patients who believed that their illness would persist for a prolonged duration experienced more distress.
Moon *et al*. [37]	Prospective cohort study on QoL of patients with low-risk papillary thyroid microcarcinoma undergoing active surveillance (n= 674) or immediate surgery (n= 381).	South Korea	Patients who underwent active surveillance had better QoL overall than those who had immediate surgery. Among those who chose immediate surgery, those who had lobectomy/ isthmusectomy had a better QoL than those who had total thyroidectomy. A higher QoL score was associated with older and male patients.	**Socioeconomic factors** Age and Sex: Older age and male sex associated with higher QoL **Management factors** Active surveillance: associated with higher QoL compared to surgery; Lobectomy: associated with better QoL than thyroidectomy.
Wang [38]	Cross-sectional study on QoL in TC patients (n=1743) using the PROMIS-29 questionnaire.	USA	Risk factors for worse QoL were: age <45 years, postoperative hypocalcemia, dysphonia, dysphagia, scar appearance & presence of short or long-term complications due to RAI treatment.	**Socioeconomic factors** Age: younger age (<45) associated with decreased QoL **Management factors** RAI: associated with lower QoL
Bresner *et al*. [39]	Cross-sectional study, quantifying cancer-related worry in TC patients (n=941), using the ASC questionnaire.	Canada	Younger TC survivors and those with confirmed or recurrent/persistent disease activity experienced the highest levels of cancer-related worry. Time since thyroid cancer diagnosis (≤ 5 years) and partnered marital status were also associated with increased worry. RAI was not significantly associated with worry.	**Socioeconomic factors** Age: younger patients at higher risk of cancer-related worry; Family status: partnered marital status at higher risk of cancer-related worry. **Disease-specific factors** Current suspected or proven recurrent/persistent disease: associated with increased worry
Smith *et al*. [40]	Cross-sectional study on psychosocial issues faced by TC patients (n=8) using semi-structured interviews.	UK	Young TC patients experienced biographical disruption, feelings of isolation, vulnerability, and being disregarded. Due to RAI treatment, they felt dirty due to compulsory glove-wearing and showering three times a day, and felt different from other cancer patients.	**Socioeconomic factors** Age: Younger patients have a more negative psychosocial impact than older patients **Patients’ perceptions** Biographical disruption; Feelings of isolation; Vulnerability and being disregarded; Feeling dirty (associated with RAI treatment).
Vega-Vázquez *et al*. [41]	Cross-sectional study of treatment effects on QoL in 75 DTC patients (n=75) using the University of Washington QoL questionnaire.	Puerto Rico	Most patients reported the same or better QoL after treatment, although this effect was not pronounced. Patients diagnosed at ≥45 years of age had better scores in the pain domain than those diagnosed at an earlier age.	**Socioeconomic factors** Age: Diagnosis at ≥45 years of age associated with better scores in the pain domain
Ramin *et al*. [42]	Prospective cohort study of RAI induced QoL changes in TC (N=132) patients using the EORTC QLQ-C30 & EORTC QLQ-H&N35 questionnaires.	Brazil	TC patients reported significant negative QoL impacts of RAI which included: nausea, vomiting, pain, sensitivity, problems with social contact, dry mouth, and sticky saliva. Global HRQoL scores improved due to RAI, as well as emotional and cognitive function. Females experienced more nausea and vomiting, and patients <55 years experienced greater sensitivity.	**Socioeconomic factors** Age: patients <55 years’ experience increased sensitivity; Sex: female patients experience more nausea and vomiting. **Management factors** RAI: may affect HRQoL negatively, yet patients may experience an improvement in global HRQoL post-therapy.
Kurumety *et al*. [43]	Retrospective study on neck appearance of TC survivors (n=1710) and subsequent QoL, using the PROMIS-29 QoL survey.	USA	Older age (>45) and longer time since surgery (>2 years) correlated with a better perception of neck appearance. Worse perception of neck appearance correlated with poorer QoL. More than 2 years after surgery, the perception of neck appearance returned to the pre-operative baseline.	**Socioeconomic factors** Age: older age associated with a better perception of appearance **Disease-specific factors** Time since surgery: longer time (>2 years) correlated with a better perception of neck appearance **Patients’ perceptions** Worse perception of neck appearance: correlated with poorer QoL
Hossain *et al*. [44]	Cross-sectional study on QoL of 246 TC (n=246) patients using interviews and an 1127-item semi-structured questionnaire.	Bangladesh	Students and graduates had the highest QoL scores out of all groups when the cohort was split by occupation and education respectively. Independent predictors of QoL were found to be education, family income, marital status, clinical condition, and perceived stress.	**Socioeconomic factors** Education: higher education or being a student associated with increased QoL; Financial status: higher family income associated with decreased QoL; Family status: being married associated with decreased QoL. **Disease-specific factors** Clinical condition: worse clinical condition associated with lower QoL scores **Patients’ perceptions** Perceived stress: more perceived stress associated with lower QoL scores
Li *et al*. [45]	Prospective observational study of post-thyroidectomy TC patients (n=286) using the EORTC QLQ-C30 questionnaire.	China	The main risk factors for decreased QoL three months after thyroidectomy were clinical stage, type of surgery, tumor histological characteristics, neurological deficits, and being single as their marital status.	**Socioeconomic factors** Family status: being single associated with decreased QoL **Disease-specific factors** Cancer stage: Higher cancer stages associated with decreased QoL; Tumor histology: papillary subtype associated with higher QoL than other subtypes. **Management factors** Type of surgery: Lobectomy associated with higher QoL than total thyroidectomy
Chen *et al*. [46]	Cross-sectional study of QoL and risk perception of recurrence and death in 2632 DTC patients using interviews with set questions and the PROMIS QoL questionnaire.	USA	Lower educated patients were more likely to overestimate the risk of recurrence and mortality compared to those with college experience. Hispanics also overestimated the risk of recurrence. Those who over-estimated also had greater worry about recurrence and death, which was also associated with a decreased QoL.	**Socioeconomic factors** Education: lower education associated with overestimation of the risk of recurrence and mortality; Ethnic background: may affect the perception of the disease. Hispanics overestimated the risk of recurrence. **Patients’ perceptions** Overestimation of risk of recurrence: associated with decreased QoL
Ye *et al*. [47]	Cross-sectional study on preoperative anxiety/ depression of TC patients (n=111). Questionnaire: HADS.	China	Significant risk factors for preoperative anxiety and depression include higher educational level (high school and above), paying for the treatment at their own expense, and poor quality of sleep.	**Socioeconomic factors** Education level: higher educational level (high school and above) associated with increased preoperative anxiety and depression; Payment for one's own treatment: associated with increased preoperative anxiety and depression.
Mongelli *et al*. [48]	Cross-sectional study of the financial burden and QoL in TC patients (n=1743). Questionnaire: PROMIS-29.	USA	Financial problems and living in poverty were associated with worse anxiety/depression in TC patients. Lost productivity at work was associated with worse fatigue & social functioning. The inability to change jobs was associated with worse fatigue, pain, and decreased social functioning. Those receiving disability benefits reported worse pain interference.	**Socioeconomic factors** Financial status: financial problems and living in poverty associated with increased anxiety and depression; Loss of productivity at work: associated with worse fatigue and social functioning.
Chan *et al*. [49]	Cross-sectional study of risk factors associated with QoL in DTC patients (n=613) using the EORTC QLQ-C30 and THYCA-QoL questionnaires.	Hong Kong	Fatigue and insomnia are the two common symptoms experienced by DTC patients. Poorer QoL was associated with the following: serum thyrotropin (TSH) of more than 1.0 mIU/L, unemployment, and concomitant psychiatric disorders.	**Comorbidities** Psychiatric illness: associated with lower QoL **Socioeconomic factors** Unemployment: associated with lower QoL **Treatment-related factors** High TSH levels: associated with lower QoL
Papaleontiou [50]	Cross-sectional study on QoL in TC patients (n=1743). Questionnaire: PROMIS-29.	USA	Higher levels of financial distress and being diagnosed for <5 years were related to worse QoL in all 7 domains. In TC survivors, employment status was an independent risk factor for QoL, with those unable to get a new job or change jobs also reporting worse fatigue, pain, and social functioning.	**Socioeconomic factors** Financial status: financial distress associated with decreased QoL; Employment status: being unable to acquire or change jobs associated with decreased QoL. **Disease-specific factors** Time since diagnosis: shorter time (<5 years) since diagnosis associated with decreased QoL
Hedman *et al*. [51]	Cross-sectional study, assessing QoL in 279 TC patients (n=279), 14-17 years after diagnosis. The SF-36 questionnaire was used in patients with and without subsequent thyroid symptoms, to compare HRQoL between the two groups.	Sweden	Patients who reported at least one physical symptom (e.g. fatigue, irritability, muscle weakness, sweating, flushes, etc.) had significantly lower HRQoL in all of the SF-36 domains, compared to patients with no symptoms.	**Disease-specific factors** Physical Symptoms: Presence of thyroid-related symptoms relates to lower HRQoL
Büttner *et al*. [52]	Cross-sectional study on TC patients (n=89) with or without hypoparathyroidism. Questionnaire: EORTC QLQ-C30.	Germany	Patients with hypoparathyroidism after TC treatment had significantly lower QoL compared to those without.	**Disease-specific factors** Patients with hypoparathyroidism after treatment at increased risk of lower QoL
Badihian *et al*. [53]	Prospective cohort study, in which 11 male (n=11) and female (n=18) DTC patients were included to determine QOL, anxiety, and depression, both during treatment with levothyroxine and during withdrawal from levothyroxine. Questionnaires: WHOQOL-Bref, BDI-II and HADS.	Iran	QoL in patients decreased after short-term hypothyroidism following withdrawal, in terms of physical and psychological health. Patients also became significantly depressed and anxious after levothyroxine withdrawal.	**Management factors** Levothyroxine withdrawal: induced hypothyroidism associated with decreased QoL
Giani *et al*. [54]	Cohort study on radioiodine-refractory DTC patients (n=39), evaluating the safety and QoL change over 6 months of Lenvatinib treatment. QLQ-C30, VAS, and EORTC determined QoL at different time points.	Italy	There were no statistically significant differences in the QoL of patients before, during, or at the end of the 6 months. During the 6 months, there was a small improvement in the general health, emotional and cognitive status but a worsening of the physical role and social functioning. Lenvatinib was also associated with side effects of fatigue, anorexia/weight loss, and stomatitis.	N/A
Banihashem *et al*. [55]	Prospective study assessing QoL in females (n=121) and males (n=29) with DTC before, during, and after treatment with RAI. HADS and SF-36 questionnaires were used.	Iran	QoL became increasingly better in patients over several months after treatment with RAI, although there was no significant difference before and during RAI treatment. The role of age, gender, RAI dose, and thyroid-stimulating hormone level at the time of RAI, showed no statistical significance.	**Management factors** RAI: associated with improved QoL afterward.
Buchmann *et al*. [56]	Retrospective cohort study evaluating levels of distress and its contributing factors in TC patients. Univariate and multivariate analyses were conducted on newly diagnosed TC (n=118) patients.	USA	43.3% of patients had significant distress. Self-reported psychiatric history, use of antidepressant medication, and history of radioactive treatment had higher levels of distress.	**Management factors** Use of radioactive treatment: associated with increased distress **Comorbidities** Psychiatric history and use of antidepressants: associated with increased distress
Ahn *et al*. [57]	Cross-sectional study comparing QoL, fear of progression, and general health between patients that underwent total thyroidectomy (TT) + radioactive iodine ablation (RAI) treatment (n=182 patients) and patients that only underwent TT (n=107). SF-12, THYCA-QoL, and FoP questionnaires were used	South Korea	Significantly lower general health was reported in patients managed with TT+RAI treatment than in patients managed with TT only. No significant difference was reported between groups for fear of progression.	**Management factors** RAI treatment: RAI + TT associated with worse QoL than TT alone
Jeon *et al*. [58]	Prospective cross-sectional study comparing QoL in PTMC patients under active surveillance (AS) (n= 43) versus those who underwent a lobectomy (LB) (n= 148). SF-12, THYCA-QoL, and fear of progression questionnaires were used.	South Korea	No significant differences in fear of progression scores between the AS and LB patients. However, LB patients experienced more health-related problems than those managed by AS.	**Management factors** Active surveillance: associated with fewer health-related problems compared to lobectomy
Nakamura *et al*. [59]	Cross-sectional study on QoL of 347 patients with low-risk papillary thyroid microcarcinoma who were under active surveillance (n= 298), or underwent immediate surgery (n= 49). Surveyed using HADS & THYCA-QoL.	Japan	Patients who underwent immediate surgery were more anxious and depressed than those who had active surveillance instead.	**Management factors** Active surveillance: associated with lower levels of anxiety and depression than thyroidectomy
Kletzien *et al*. [60]	prospective mixed-methods observational study that aimed to determine the quality of life (QoL) consequences of post-thyroidectomy voice changes in patients with papillary thyroid cancer (PTC). The study involved 42 patients and compared patient-perceived voice changes with changes in quantitative vocal variables at five different time points during the first year after surgery. The study utilized semi-structured interviews, assessments of symptom prevalence, and instrumental voice evaluations.	USA	After thyroidectomy, impaired communication was the primary theme in patient interviews--. Patients mentioned the inability to communicate and work, and feelings of frustration. The 2-week follow-up detected quantitative vocal perturbations in some patients, which returned to baseline levels by the 6-week follow-up.	**Management factors** Impaired communication following thyroidectomy: associated with frustration
Nickel *et al*. [61]	Cross-sectional study on QoL after diagnosis and treatment of DTC patients (n=1005). The study used open-ended questions to gather data on the patients' quality of life.	Australia	77% of TC patients reported experiencing QoL issues as a result of TC diagnosis and treatment. Patients who had a total thyroidectomy were more likely to have a lower QoL than those who had a hemithyroidectomy.	**Management factors** Thyroidectomy: associated with lower QoL than hemithyroidectomy
Lan *et al*. [62]	Cross-sectional study comparing the QoL in papillary thyroid microcarcinoma patients (n=69) treated with thyroidectomy versus lobectomy using the SF-36, THYCA-QOL and Fear of Progression Questionnaire-Short Form (FoP-Q-SF).	China	Results from the SF-36 questionnaire showed a linear association with worse QoL in the physical and mental domains in both the thyroidectomy and lobectomy treatment group. Results from the THYCA-QOL showed that thyroidectomy was associated with a worse scar appearance and hence QoL than lobectomy. There was no significant difference in the FoP-Q-SF results between the groups.	**Treatment-related factors** Thyroidectomy: associated with a lower QoL compared to lobectomy.
Bongers *et al*. [63]	Cross-sectional study comparing long-term health-related quality-of-life of DTC survivors treated with hemithyroidectomy (n=59) compared to survivors treated with total thyroidectomy (n=211) between 2005 and 2016. Questionnaires: THYCA-QoL, EORTC QLQ-C30 and ACS.	Canada	Long-term QoL was not significantly different between the two groups, however, secondary analyses showed hemithyroidectomy patients have more worry of recurrence (on one survey).	**Management factors** Thyroidectomy: Hemithyroidectomy associated with higher worry of recurrence, compared to total thyroidectomy
Luddy *et al*. [64]	Prospective study of QoL in TC patients (n= 182) after undergoing thyroidectomy and lobectomy using the SF-36 questionnaire.	USA	Patients with DTC had a poorer QoL than patients with benign thyroid pathology. There were no significant differences before and after surgery in the mental and physical domains of QoL between TC patients undergoing thyroidectomy and lobectomy.	n/a (no significance differences found in thyroidectomy vs lobectomy)
Kim *et al*. [65]	Cross-sectional study comparing QoL in patients receiving open (n=117) and robotic (n=112) total thyroidectomy respectively. QoL included overall satisfaction, cosmetic results, and various post-op changes.	South Korea	Overall satisfaction and cosmetic results were significantly higher in patients receiving a robotic thyroidectomy, than an open thyroidectomy. There were no other differences in the results of other variables among the two groups, concluding that a robotic thyroidectomy showed comparable results to open thyroidectomy in terms of the postoperative long-term QoL.	N/A
Song *et al*. [66]	Prospective, cross-sectional, observational study, assessing QoL of papillary TC patients (n=114) who received a transoral robotic thyroidectomy (n=57) or conventional transcervical thyroidectomy (n=57).	South Korea	QoL in relation to neck appearance was higher following transoral robotic thyroidectomy than after transcervical thyroidectomy. Total QoL scores however did not differ in the 2 groups after surgery.	**Treatment-related factors** Transcervical thyroidectomy: associated with worse perception of neck appearance than transoral robotic thyroidectomy.
Chen and Chen [67]	A retrospective cohort study comparing postoperative QoL and cosmetic outcome between minimally invasive video-assisted thyroidectomy (MIVAT) and bilateral axillo-breast approach (BABA) robotic thyroidectomy.	Taiwan	No significant difference in scar perception between the two groups. However, the MIVAT group had better post-operative aspects of QoL than the BABA robotic group such as general health, vitality, mental health, and health change.	**Treatment-related factors** Bilateral axillo-breast approach robotic thyroidectomy: associated with lower QoL compared to minimally invasive video-assisted thyroidectomy.
Lan *et al*. [68]	Cross-sectional study comparing QoL of PTMC patients (n=54) receiving RFA to PTMC patients (n=34) undergoing surgery. SF-36, THYCA-QOL, and FoP-Q-SF questionnaires were used.	China	No statistically significant difference in the scores of FoP-Q-SF, between the two groups.	N/A
Metallo *et al*. [69]	Cross-sectional study on QoL and self-esteem and pregnancy outcomes of female DTC survivors (n=45) treated with thyroidectomy and I131 before the age of 25. The SF-36 and ISP-25 questionnaires were used.	France	Young and female DTC survivors reported no long-term negative impacts of TC on their QoL, self-esteem, or pregnancy outcomes.	N/A
Wiener *et al*. [70]	Cross-sectional study of the relationship between type D personality and QoL in DTC survivors (n=284).	USA	Depression itself, and not Type D personality, was an independent predictor of QoL in DTC survivors and negatively impacted QoL in all domains.	**Comorbidities** Pre-existing depression: has a negative impact on QoL
Henry *et al*. [71]	Cross-sectional study on the experiences, preferences, and needs of TC (n=17) patients using interviews.	Canada	Uncertainty and lack of support/being overlooked due to TC’s good prognosis are recurring themes among TC patients.	**Patients’ perceptions** Fear about surgical complications, metastasis Lack of support
Randle *et al*. [72]	Prospective qualitative study on QoL of papillary TC patients (n=31) before and after thyroidectomy using semi-structured interviews.	USA	The notion that TC is a "good cancer" is widely spread throughout the healthcare system, online search results, and social circles. This had a negative impact on the patients as it invalidates their experiences and fears, and was found to be a cause of mixed/confusing emotions.	**Patients’ perceptions:** Feeling their experiences are invalidated due to TC being overly considered as a “good cancer”.
Barbus *et al*. [73]	Cross-sectional study on QoL of DTC (n=135) patients using the QoL-TV questionnaire.	Romania	Psychological well-being in DTC patients was related to treatment, diagnostic tests, and the possibility of recurrence/metastasis. No relations were found between age/gender and QoL.	**Disease-specific factors** Possibility of recurrence: related to worse psychological well-being and therefore decreased QoL **Management factors** Treatment: related to worse psychological well-being and therefore decreased QoL; Diagnostic tests: related to worse psychological well-being and therefore decreased QoL.
Hedman *et al*. [74]	Prospective population-based study, studying changes in HRQoL from diagnosis to one-year follow-up, in DTC patients (n=235). SF-36 and a study-specific questionnaire were used at the two-time points.	Sweden	HRQoL in patients was most affected at the time of diagnosis, with improvements in some patients after one year. Having a fear of recurrence and a negative view of life, were the two most influential factors affecting HRQoL negatively after one year.	**Patients’ perceptions** Fear of recurrence: associated with lower HRQoL; Negative view of life: associated with lower HRQoL.
Rogers *et al*. [75]	Cross-sectional study on QoL in DTC (n=169) patients, using the EORTC QLQ-C30, THYCA-QoL, Emotion Thermometers, and FoR questionnaires.	UK	Global health status and emotional domains in QoL were the most affected. Most problems with HRQoL include - fatigue, issues with sleep, pain, dry mouth, hot flushes, and weight gain. 1 in 7 patients stated they struggled with fear of recurrence. Distress was relatively low.	**Patients’ perceptions** Fear of recurrence: associated with decreased QoL
Hedman *et al*. [76]	Cross-sectional interview study of DTC patients (n=21) about fear/anxiety associated with recurrence.	Sweden	Anxiety in TC patients was mainly due to fear of recurrence and exhausting effective treatment options.	**Patients’ perceptions** Fear of recurrence: related to increased anxiety; Fear of lack of effective treatment options in the future: related to increased anxiety.
Schoormans *et al*. [77]	Cross-sectional study of DTC survivors (n=284) surveyed using the B-IPQ and EORTC QLQ-C30 questionnaires.	The Netherlands	QoL was poorer in DTC survivors who had the following perceptions about their illness: it had many negative consequences, it was the cause of their symptoms and negative emotions, and it could be controlled by treatment.	**Patients’ perceptions** Negative perceptions from patients: correlates with a decreased QoL.
Liu *et al*. [78]	Prospective study on the impact of scar appearance post thyroidectomy on QoL and satisfaction with aesthetic effect using the EORTC QLQ-C30 and Patient Scar Assessment Scale (PSAS).	China	QoL was inversely related to PSAS score (where higher equals a worse appearance due to scar irregularity and length)	**Patients’ perceptions** Poor satisfaction of post-surgical cosmesis: associated with lower QoL.

#### Age

Age has been associated with decreased QoL in older adults and increased worry in younger adults, although some studies have reported conflicting results. For example, older age has been associated with a decreased QoL in some studies [19, 25, 34, 35], while younger age has been associated with increased worry [34, 35]. However, other studies have reported that younger age is associated with a lower QoL in TC patients [14, 33, 37, 38]. One study [28] found that young female adults (≤44) suffering from TC had an increased incidence of depression compared to older females, whereas another study [39] showed that younger patients were at a higher risk of cancer-related worry compared to older patients. Despite the good prognosis of TC, young patients may feel dismissed and experience uncertainty about their future [40]. Older age was linked to better pain management and improved perception of appearance, which is associated with higher QoL [41-43].

#### Family status

Family status has been investigated in relation to depression, anxiety, and QoL in TC patients across several studies [15, 34, 39, 44, 45]. While being in a "partnered marital status" and "having children" were associated with increased cancer-related worry, being married was linked to decreased QoL in some studies [39, 44]. However, being married was found to be associated with a favorable QoL compared to being unmarried or divorced in other studies [15, 45].

#### Education and ethnic background

Higher educational level was associated with a higher QoL in TC survivors [15, 33, 34, 44, 46], although two studies [24, 47] reported conflicting results and a higher level of education was associated with a significantly lower QoL and increased pre-surgery anxiety and depression respectively. In terms of ethnic background, one study reported that Asian and Hispanic TC patients in the USA experienced greater worry compared to Caucasian patients [35].

#### Financial and employment status

Financial strain and employment status have also been associated with anxiety, depression, or QoL in TC [15, 24, 26, 28, 35, 44, 47-50]. Financial difficulties were reported in 43% of TC survivors, leading to higher levels of anxiety and depression, lost productivity, and an inability to change jobs [48]. Low-income TC patients experienced higher rates and longer periods of depression one year after thyroidectomy compared to higher-income patients [28]. Similarly, lower socio-economic backgrounds were linked to increased cancer-related worry and worse QoL scores [15, 35, 50]. Hossain *et al*. [44] found that higher family income was associated with decreased QoL. Unemployment significantly lowered the QoL of TC survivors in three studies [24, 26, 49], while paying for treatment out-of-pocket was associated with increased preoperative anxiety and depression in one study [47].

#### Disease-specific factors

Several studies have identified disease-specific factors associated with anxiety, depression, and low QoL in TC patients [22, 23, 26, 28, 39, 44, 45, 50, 51]. These factors include tumor TNM stage and the presence of metastases, physical symptoms, current active disease or cancer recurrence, and time since diagnosis and treatment. For example, a higher tumor TNM stage and the presence of metastases have been associated with a lower QoL in some studies [22, 25, 45]. Physical symptoms have also been found to be a significant predictor of lower QoL in TC patients [44, 51]. Moreover, TC patients with current active disease or cancer recurrence experienced more cancer-related worry compared to those in remission [39]. Another disease-related risk factor for TC patients is the time since diagnosis and treatment. Several studies [23, 43, 50] reported that a longer time since diagnosis was associated with a better QoL. In addition, TC patients in the later stages of survivorship (>5 years) had less cancer-related worry than those in the earlier stages [39]. Consistently, Choi *et al*. [28] reported that TC survivors had a significantly higher incidence of depression in the first postoperative year compared to matched controls.

#### Management factors

The impact of different management factors on the QoL, anxiety, and depression of thyroid cancer (TC) patients has been explored in 28 studies reviewed in this study [14, 19-22, 26, 29, 37-40, 45-49, 52-69].

#### Hypothyroidism, Levothyroxine withdrawal, and Lenvatinib

The development of hypothyroidism and hypoparathyroidism after treatment of TC has been associated with a significantly worse QoL score [14, 52]. Withdrawal of levothyroxine, a medication used to treat hypothyroidism, is also a risk factor for decreased QoL [19, 53]. Conversely, exogenous TSH stimulation was associated with improving certain aspects of QoL [21], while higher TSH levels were associated with a lower QoL [49].

#### Radioactive iodine treatment

Radioactive iodine treatment (RAI) has been extensively studied as a predictive and protective factor of QoL among TC patients. While some studies have reported improvements in QoL and emotional and cognitive function after RAI treatment, patients have also reported significant side effects such as nausea, vomiting, and RAI-induced xerostomia [29,42] that negatively impact QoL. Banihashem *et al*. [55] discovered that QoL only improved several months after RAI treatment, whereas some studies provided evidence that RAI had a negative impact on QoL [14, 22, 38, 56, 57]. One study found that TC patients receiving RAI treatment did not have significantly different levels of worry compared to those not receiving RAI [39]. Patients undergoing RAI reported feeling isolated and dirty due to the compulsory glove-wearing and showering three times a day and perceived themselves as different from other cancer patients [40].

#### Active surveillance, lobectomy, and thyroidectomy

Active surveillance as an alternative to surgery is associated with a higher QoL [37, 58] and lower levels of anxiety and depression compared to thyroidectomy [59]. Complications arising from thyroidectomies, such as neurological deficits resulting from nerve damage [45] and impaired communication, a primary complaint among TC patients and a significant source of frustration [60], may explain the negative impact of surgery on QoL outcomes.

Research suggests that QoL differs among TC patients who undergo lobectomy, hemithyroidectomy, or total thyroidectomy. Four studies [37, 45, 61, 62] reported that lobectomy was associated with a better QoL than thyroidectomy. In contrast, one study [63] reported that hemithyroidectomy was associated with a higher worry of recurrence compared to thyroidectomy, whereas Luddy *et al*. [64] reported no significant differences in the mental and physical domains of QoL in patients who underwent thyroidectomy compared to patients that underwent lobectomy.

#### Other surgical approaches

Two studies found no significant differences in QoL between patients who underwent transoral robotic thyroidectomy and those who underwent conventional open thyroidectomy [65, 66]. A retrospective study [67] found that patients receiving minimally invasive video-assisted thyroidectomy had better QoL outcomes compared to the bilateral axillo-breast approach robotic thyroidectomy. Furthermore, Lan *et al*. [68] reported no significant differences in QoL scores between patients who received radiofrequency ablation (RFA) and those who underwent surgery. Finally, Metallo *et al*. [69] carried out a study on female AYA DTC survivors who underwent thyroidectomy and RAI and reported no negative long-term impacts on their QoL, self-esteem, or pregnancy outcomes.

### Comorbidities

Several studies have reported a positive association between comorbidities and anxiety, depression, or lower QoL in TC survivors [17, 20, 24, 30, 32, 34, 49, 56, 70].

### Patient perceptions

The association between patients’ perceptions and anxiety, depression, or QoL in TC has been reported in 16 studies [17, 34-36, 40, 43, 44, 46, 71-78]. Patients’ worries over recurrence were linked to impaired QoL [35]. According to Henry *et al*. [71], common sources of fear for patients with TC included surgical complications, metastasis, and a perceived lack of support due to feeling overlooked by the healthcare system.

Randle *et al*. [72] reported that TC patients were aware of how TC is thought of as a “good cancer” by medical professionals, their peers, and internet websites, which resulted in their fears feeling invalidated. Smith *et al*. [40] reported that although younger TC survivors understood the concept of a “good cancer,” this did not offset the negative aspects they experienced. The study identified the common theme of “biographical disruption,” where survivors struggled with a loss of youthful immunity, uncertainties about the future, fears of recurrence, and feelings of being disregarded, vulnerable, and isolated.

Several studies have demonstrated that fear of recurrence and a negative outlook on life is linked to decreased quality of life (QoL) among individuals with TC [17, 34, 73-75]. Chen *et al*. [46] reported that patients who overestimated their risk of cancer recurrence also worried more about death and reported a lower QoL. Hedman *et al*. [76] found that fear of recurrence and fear of exhausting effective treatment options in the future were related to increased anxiety levels. In addition, several studies [36, 44, 77] showed that perceived stress, or having more negative perceptions of cancer, was associated with lower QoL. Kurumety *et al*. [43] found that patient perceptions regarding neck appearance after surgery were also associated with changes in quality of life (QoL). The study showed that patients over 45 and those who had undergone surgery more than two years before reported better-perceived neck appearance and QoL than those with more recent surgery. However, after two years, the perceptions of TC patients regarding neck appearance had fully returned to the pre-operative baseline. These findings suggest that while cosmetic appearance may impact QoL, its effect may be lost in the long term.

### Protective factors associated with anxiety, depression, and QoL in TC patients

The association between protective factors and anxiety, depression, or QoL in TC has been reported in 3 studies (Table 4) [79-81]. One prospective study [79] established that patients who received more dedicated time for information-giving were less anxious about the treatment and concluded that good communication between the patient and the medical team is associated with increased QoL. A randomized control trial by Wang *et al*. [80] assessed TC patients who received the psychological nursing intervention compared to TC patients who did not and reported that patients who received the nursing intervention had significantly lower levels of depression, anxiety, and mood disturbances compared to the control. Similarly, Wu *et al*. [81] reported that psychological nursing interventions resulted in greater improvements in QoL, depression, and anxiety compared to conventional nursing.

**Table 4:** Studies examining protective factors associated with lower anxiety and depression and higher QoL in thyroid cancer patients.

Study	Study details (design, population and methods)	Country study was conducted	Key findings	Protective factors categories
Barbus *et al*. [79]	Prospective study of 51 DTC patients (n=51) using questionnaires to determine psychological impact before and after radioiodine treatment.	Romania	Pre-treatment patients had good confidence in the treatment and said the information given was accurate, but >50% had anxiety. Post-treatment patients had less anxiety, and would do it again or recommend it to others. More time dedicated to information giving was associated with less anxiety experienced regarding treatment.	**Good communication between the patient and the medical team:** associated with increased QoL
Wang *et al*. [80]	Randomised controlled trial on the impact of psychological nursing intervention on QoL of TC patients (n=286). 143 participants in both intervention and control groups, with QoL, assessed using the EORTC QLQ-C30 questionnaire.	China	Patients in the nursing intervention group had significantly lower levels of depression, anxiety, and mood disturbances.	**Psychological nursing interventions:** may reduce depression anxiety and mood disturbances
Wu *et al*. [81]	Randomised controlled trial of DTC patients post-RAI treatment (n=60), undergoing either conventional nursing (n=30) or psychological and behavioral intervention-based nursing (n=30). QoL, anxiety, and depression were respectively measured using the Quality of Life Core Questionnaire, Self-rating Anxiety Score, and Self-rating Depression Score.	China	QoL and functional capacities of both groups improved after RAI treatment. At 1-year follow-up, patients in the psychological and behavioral intervention group had more improvement in QoL scores, anxiety, and depression compared to those in the conventional nursing group.	**Psychological and behavioral nursing interventions:** associated with greater improvement in QoL, anxiety, and depression outcomes than conventional nursing

## DISCUSSION

The current scoping review was conducted to investigate the effects of TC on anxiety, depression, and QoL of patients and to examine the various risk factors associated with lower QoL in TC patients. Overall, TC survivors were found to have a worse QoL compared to the general population [14, 16-18, 20-22, 24-29], patients with benign thyroid pathology, and even survivors of other cancer types with worse prognoses [30-33].

**Figure 2:**
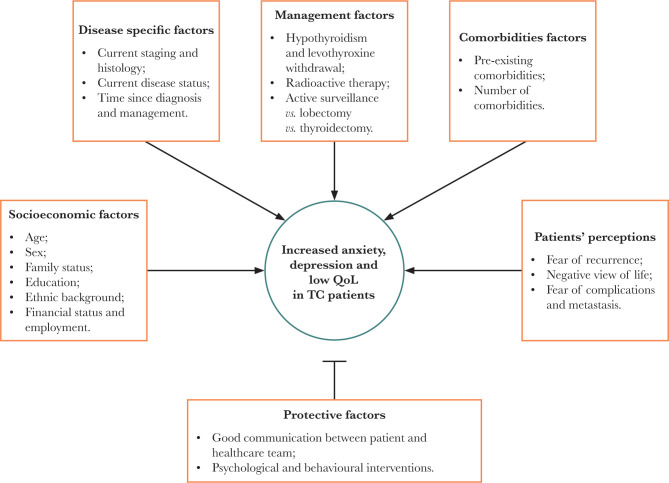
The factors associated with increased anxiety and depression and decreased QoL can be grouped into socioeconomic factors, disease-specific factors, management factors, comorbidities, and patient perceptions. Protective factors include good communication between the health care team and patients and psychological and behavioral interventions. TC – thyroid cancer.

The factors associated with increased anxiety and depression and decreased QoL were grouped into socioeconomic factors, disease-specific factors, management factors, comorbidities, and patient perceptions (Figure 2).

Most of the studies supported that a younger age at diagnosis as well as being female is generally associated with increased anxiety, depression, and a worse QoL [14, 24, 28, 33, 43]. Some studies reported that being married/having a partner was a protective factor for a higher QoL [15, 35, 45], and others supported it as a risk factor for poorer QoL [39, 44]. In addition, there was significant evidence that higher education plays a favorable role in TC survivors’ QoL and anxiety [15, 33, 34, 44, 46]. However, some studies found that higher levels of education were associated with lower QoL and more anxiety [24, 47]. Studies also reported that financial difficulties were associated with a lower QoL and/or more distress in TC patients [15, 24, 26, 35, 47-50].

Our paper reports that disease-specific factors such as active disease [39, 44, 51] and shorter length of time since diagnosis or treatment [23, 28, 39, 43, 50] are also associated with increased risk of anxiety, depression, and QoL in TC patients. Furthermore, the side effects of treatment [14, 52, 53] and the type of treatment were also identified as important factors affecting the QoL [37, 45, 61, 62]. In addition, active surveillance rather than surgery had better outcomes [37, 58, 59]. Having comorbidities increased stress and anxiety, leading to a poorer QoL [17, 20, 24, 30, 34, 49, 55, 70], whereas the patients’ perceptions related to diagnosis, treatment, and neck-scarring post-treatment were also associated with increased anxiety and depression and decreased QoL [35, 36, 43, 44, 71, 72, 77, 78].

Our study supported some protective factors associated with increased QoL, which include good communication between the patient and the healthcare team via adequate and accurate information-giving, which can decrease patients’ anxiety levels and improve their QoL [79]. Psychological and behavioral interventions were also shown to improve QoL and reduce depression and anxiety [80, 81].

The findings of our scoping review indicate that there are still several gaps in our understanding of the effects of TC on the QoL, anxiety, and depression of patients. To address these gaps, future research should focus on conducting more rigorous studies, such as randomized controlled trials, to thoroughly assess the risk factors identified in this review. Additionally, qualitative research could help provide further insights into how both risk factors and protective factors impact the patients' experience of TC. Once the risk and protective factors are appropriately assessed, the emphasis could shift towards a more interventional and holistic approach for managing TC patients and survivors. Healthcare professionals should be educated on the risk factors associated with lower QoL in TC survivors, and studies could be designed on interventions specifically targeted toward improving QoL. It is expected that these studies could involve the development of successful interventions that could be incorporated into the routine management of TC patients.

## CONCLUSION

TC is often overlooked as a "good cancer", which is not only a misconception given its strong association with anxiety, depression, and poor QoL but also undermines the experiences of patients and survivors. Nevertheless, TC patients and survivors face numerous risk factors associated with higher levels of anxiety and depression and lower QoL. Furthermore, limited evidence supports good communication between the patient and the healthcare team, and psychological and behavioral interventions may protect the patients from anxiety, depression, and low QoL. Therefore, TC policy survivorship programs should involve the education of multi-professional healthcare teams on the various risk factors associated with the development of anxiety, depression, and low QoL in TC patients. A holistic and multi-level approach is necessary to address the various challenges associated with this "not so good cancer", which should aim to address TC patients' concerns and enhance their quality of life during the survivorship phase.
